# A call for differentiated approaches to delivering HIV services to key populations

**DOI:** 10.7448/IAS.20.5.21658

**Published:** 2017-07-21

**Authors:** Virginia Macdonald, Annette Verster, Rachel Baggaley

**Affiliations:** ^a^ HIV Department, World Health Organisation, Geneva, Switzerland

**Keywords:** differentiated care, antiretroviral therapy, HIV testing services, PrEP, key populations

## Abstract

**Introduction**: Key populations (KPs) are disproportionally affected by HIV and have low rates of access to HIV testing and treatment services compared to the broader population. WHO promotes the use of differentiated approaches for reaching and recruiting KP into the HIV services continuum. These approaches may help increase access to KPs who are often criminalized or stigmatized. By catering to the specific needs of each KP individual, differentiated approaches may increase service acceptability, quality and coverage, reduce costs and support KP members in leading the HIV response among their communities.

**Discussion**: WHO recommends the implementation of community-based and lay provider administered HIV testing services. Together, these approaches reduce barriers and costs associated with other testing strategies, allow greater ownership in HIV programmes for KP members and reach more people than do facility-based services. Despite this evidence availability and support for them is limited.

Peer-driven interventions have been shown to be effective in engaging, recruiting and supporting clients. Some programmes employ HIV-positive or non-PLHIV “peer navigators” and other staff to provide case management, enrolment and/or re-enrolment in care and treatment services. However, a better understanding of the impact, cost effectiveness and potential burden on peer volunteers is required.

Task shifting and non-facility-based service locations for antiretroviral therapy (ART) initiation and maintenance and antiretroviral (ARV) distribution are recommended in both the consolidated HIV treatment and KP guidelines of WHO. These approaches are accepted in generalized epidemics and for the general population where successful models exist; however, few organizations provide or initiate ART at KP community-based services.

**Conclusions**: The application of a differentiated service approach for KP could increase the number of people who know their status and receive effective and sustained prevention and treatment for HIV. However, while community-based and lay provider testing are effective and affordable, they are not implemented to scale. Furthermore regulatory barriers to legitimizing lay and peer providers as part of healthcare delivery systems need to be overcome in many settings. WHO recommendations on task shifting and decentralization of ART treatment and care are often not applied to KP settings.

## Introduction

Key populations (KPs) – a broad grouping of people that includes people who inject drugs, sex workers, men who have sex with men, transgender people and people living in prisons and closed settings – are disproportionately affected by HIV epidemics. People who inject drugs and men how have sex with men are up to 24 times more likely and sex workers are up to 10 times more likely to acquire HIV than adults in the general population [1]; transgender women are 49 times more likely to be living with HIV than other adult females [2]; and almost 4% of all people incarcerated are estimated to be living with HIV [3].

A range of structural factors stemming from the criminalization of behaviours compounds KP members’ vulnerability and leads to their stigmatization and exclusion from health services. Drug use is criminalized in most countries and the “global war on drugs” continues to fuel negative stereotypes of drug users. Consensual, same-sex sex between adults is punishable by imprisonment in 73 countries and the death penalty in 13 [4]; some aspect of sex work is criminalized in more than 100 countries [5]. One in five men who have sex with men in Botswana, Malawi, Namibia and South Africa report that they are afraid to seek health services; almost half had experienced human rights abuses [6]. A survey of almost 2000 female sex workers in Cameroon found that most had experienced violence and this was associated with fear of health services and mistreatment in a health centre, as well as inconsistent condom use [7].

For these reasons: criminalization, violence, human rights abuses, stigma and discrimination, there is a lack of availability of high-quality KP-focused HIV prevention, testing, treatment and care services, and KP members are often excluded from facility-based HIV testing services and antiretroviral therapy (ART). The uptake of HIV testing among KP is low: globally, in 2015, an average of 49% of men who have sex with men, 42% of people who inject drugs and 56% of sex workers had received an HIV test and knew the results in the last one year [8]. While data are limited, reports to UNAIDS indicate an average of 24% of people who inject drugs, 21% of sex workers and 14% of men who have sex with men were enrolled on ART in 2015 [8]; these are in contrast to UNAIDS estimates of global coverage of ART at 46% in 2015 [9].

Differentiated HIV service delivery is a client-centred approach that aims to maximize the reach, quality, effectiveness, efficiency and impact of HIV services and resources. Key elements of differentiated care include service frequency, service intensity, service location and health worker cadre with these components combined differently depending on country, population and individual clients’ clinical and social needs. The aim is improvement of patient care and reductions in patient load for facility-based services with decreasing opportunity costs and increasing acceptability for clients [10]. While most frequently referred to in relation to ART provision in generalized epidemic settings, using differentiated approaches for KP-targeted HIV testing, treatment and prevention (including provision of pre-exposure prophylaxis (PrEP)) services is also promoted by WHO and others. These approaches could provide particular benefits such as reaching marginalized, criminalized and stigmatized populations, increasing acceptability, quality and coverage of testing and treatment programmes and supporting KP members in leading the HIV response among their communities.

## Discussion

### Task shifting and changing service locations: lessons from testing and treatment policy and implementation

Services for KPs have long-employed different delivery approaches, including providing services on outreach, community empowerment, employing and supporting peers and outreach workers, implementing community-based programmes and the provision of an integrated and comprehensive package of HIV, health and social services. These are evidence-based approaches which are recommended by WHO [11].

The WHO consolidated guidelines on HIV testing services recommend implementation of community-based HIV testing services and for lay providers, including KP members, to perform HIV tests. This includes using the “test for triage” approach where a single rapid test is performed in a community setting and those with a reactive test supported to link for further testing at a facility [12]. In addition, HIV self-testing (HIVST) guidelines from WHO were launched in December 2016. Together, these approaches reduce barriers and costs associated with other testing strategies, allow greater ownership and involvement in HIV services for KP members and reach more people, often at an earlier stage of the disease than do facility-based services [13]. However, the availability of HIVST is currently limited largely to demonstration projects and many countries, particularly those with concentrated epidemics, still do not support lay provider and peer testing despite WHO recommendations. Delays in adopting these approaches constitute barriers to supporting HIV diagnosis for KP members.

While expanded testing approaches which lead to increased service responsibility and involvement for community-based organizations and peers are gaining support, the role of KP community-based organizations and members in ART initiation and ARV distribution has garnered less attention. Further, while task sharing and non-facility-based service locations for ART initiation and maintenance and ARV distribution are accepted in generalized epidemics and for the general population where successful models exist [14], few organizations provide or initiate ART at KP community-based services. Some examples include The Liverpool Voluntary Counselling and Testing Health Key Populations programme in Nairobi, Kenya, which provides men who have sex with men and sex workers with a comprehensive package of HIV services in health facilities and through outreach covering the entire testing and treatment continuum including the delivery of ART [15]; Sisters Antiretroviral therapy Programme for Prevention of HIV – Integrated Response in Zimbabwe, which previously provided onsite ART for sex workers (but had to end ART provision service for lack of funding); and Médecins du Monde in Myanmar, which provides comprehensive services for people who inject drugs, including opioid substitution therapy (OST) and ART. However, in general, KP services that provide ART on site in low-middle income countries are rare and/or undocumented.

The initial impetus for the differentiated care approach in generalized epidemic settings was to respond to overburdened national ART programmes and clinicians, particularly in light of increasing numbers of people requiring treatment given the adoption of a treat all policy in many countries. For KP, this approach brings the additional potential advantage of addressing exclusion from facility-based ART services. Studies suggest that KP members feel more comfortable accessing services at community-based sites and from peers or other sensitized healthcare workers [16–18], and experience from HIV testing services shows that it is favourable and possible to share tasks with KP members [19].

All WHO recommendations supporting differentiated care apply to KPs. For example, WHO recommendations that ART can be initiated and/or maintained in peripheral health facilities can extend to KP community-health services; the WHO recommendation that trained and supervised lay providers can distribute and dispense ART can apply to trained and supported KP peers. Also trained and supervised community-health workers can dispense ART between visits to KP members through community-based services [11,20]. Specifically for opioid-dependent drug users, improved ART adherence can be achieved through provision of OST and integration of ART, OST and tuberculosis (TB) services [21]. While these approaches are recommended in both the consolidated HIV treatment and KP guidelines of WHO, uptake in low- and middle-income countries is slow.

A similar approach of differentiated service delivery of PrEP may be considered for KPs. PrEP is recommended by WHO for people at substantial and ongoing HIV risk. In many settings men who have sex with men, transgender women and sex workers may benefit from the offer of PrEP services. As for ART, developing ways to provide community delivery and support for adherence tailored to needs of people taking PrEP will need to be developed if PrEP services are to be acceptable and effective.

### Intensive service provision: case management and peer navigation

Low levels of ART literacy, inexperience navigating hospital- and clinic-based services, stigma, discrimination and fear of identifying or describing criminalized behaviours may dissuade KP members from seeking and receiving HIV treatment and other health services. Often, the first point of contact for KP members are community-based and peer-led services, either through outreach or at strategically located drop-in centres. The idea of employing peers to provide assisted referrals for KP members (i.e. accompanying people to HIV service appointments) and peer support for adherence and retention in HIV services is not new. Increasingly, KP services also employ “peer navigators” and other staff to provide case management support beyond just referral for KP members newly enrolled on ART programmes [22,23]. For example, the HIV Foundation of Thailand, which provides support to newly diagnosed men who have sex with men and other vulnerable groups in Bangkok, utilizes a traffic light system whereby high needs, or “red light” clients (i.e. with CD4 ≤ 200 and not yet taking ART), receive intensive support such as peer accompaniment to hospital appointments and linking to other welfare and social services. For “green light” clients, that is those with CD4 ≥ 500, initial intensive support is provided to ensure CD4 is subsequently measured and support timely ART initiation; once stable these clients are supported by phone [24].

Little evidence exists on evaluating the effectiveness of case management or peer navigation approaches for improving KP retention in ART programmes and/or viral suppression. A randomized control trial found intensive case management plus financial incentives were not shown to increase rates of viral suppression in people who inject drugs [25], but other positive outcomes of peer navigation [26] or case management [27–29], such as linking and retaining people in other treatment, health and social welfare programmes and improving individual functioning are frequently reported.

As programmes move increasingly to case management and peer navigation approaches for KPs, cost effectiveness will need to be assessed. The burden on already overloaded peers who often work as volunteers is also a consideration and models to compensate workers must be assessed and implemented.

## Conclusions

Before the 2020 HIV targets (90% of all PLHIV know their HIV status; 90% of all people with diagnosed HIV infection receive sustained ART, and 90% of all people receiving ART have viral suppression) can be met, many more KP members need to know their HIV status, access appropriate prevention services, receive and be retained on ART to achieve viral suppression; without their inclusion across the cascade, we will not achieve epidemic control. Currently, KP members are under-represented in treatment populations and too many are unaware of their status. Clearly, new approaches are needed.

The application of a differentiated service approach for KP could increase the number of people who know their status and receive effective and sustained prevention and treatment for HIV. However, while community-based and lay provider testing are effective and affordable, they are not implemented to scale, and regulatory barriers to legitimizing lay and peer providers as part of healthcare delivery systems need to be overcome in many settings. WHO recommendations to task shift and decentralize ART treatment and care are often not applied to KP settings. Effective models do exist for the general population and, to a lesser extent, for KP, and these should be used as a starting point for planning and advocacy for a new approach to testing and treating KP members.
